# New Arrangement

**Published:** 1872-11

**Authors:** 


### EDITORIAL AND MISCELLANEOUS.

---

New Arrangement.—Under a recent arrangement, *The Atlanta Herald Publishing Company* become the proprietors of the Atlanta Medical and Surgical Journal.

The change in the publication office involving negotiations and special arrangements has necessarily caused delay in the issue of the November number. It is designed to issue the December number in a few days following the present number, and the January number by the 10th of that month; and the succeeding numbers not later than the 10th of each month.

The new proprietors, having investigated the status and prospects of the Journal, and having become satisfied of the value of the enterprise in a business point of view, are determined to spare no effort to increase, in every possible way its merits, and to furnish a journal which, it is hoped, will be appreciated by the entire profession.

This Journal is now in its tenth volume, aud is permanently established, and would be continued without any addition to its present revenue, but the new publishers are not satisfied with anything less than "Excelsior," in anything they undertake, and their intention is to infuse all the'energy and enterprise into the publication which has carried the *Daily Herald* (of which they are the proprietors) so rapidly to the front rank of journalism. Besides the already large list of eminent contributors and collaborators, there will be constant additions to the sources from which we shall derive original matter for the Journal, and special arrangements will be made for furnishing the best materials (in an attractive form) which can be found in the Foreign Journals.

We congratulate the subscribers to the Journal upon the change which has been made in its business arrangements. With very little effort the Journal has been put upon a substantial financial basis, and under the new arrangement, and with the determination upon the part of the proprietors to spare neither labor nor expense in making it worthy of a largely extended patronage, we shall hope to be in communication with a very much larger number of our medical friends.

For ourselves, and others who will be associated with us in the future, we can only promise renewed efforts to furnish a Journal of the highest order.

